# Clinical Pharmacist Recommendations for Rational Benzodiazepine and Zolpidem Use During Daily Ward Rounds in Inpatients with Mental Disorders: A Retrospective Pre-Post Study

**DOI:** 10.1192/j.eurpsy.2025.2100

**Published:** 2025-08-26

**Authors:** M. Stuhec, A. G. Gazdag, Z. Cuk, R. Oravecz, B. Batinić

**Affiliations:** 1 Department of Pharmacology, University of Maribor, Medical Faculty Maribor; 2 Department of Clinical Pharmacy, Ormoz’s Psychiatric Hospital, Maribor; 3 Clinical Pharmacy; 4 Ormoz’s Psychiatric Hospital, Ormoz, Slovenia; 5 Department of Psychology, University of Belgrade, Faculty of Philosophy, Belgrade; 6 Clinic of Psychiatry, University Clinical Centre of Serbia, Belgra, Serbia

## Abstract

**Introduction:**

Benzodiazepines and zolpidem are commonly prescribed as long-term treatments for anxiety and insomnia, although recommendations generally do not support their extended use. These medications are often prescribed for longer durations than necessary, at inappropriate doses, and with potential drug-drug interactions. In this context, rational prescribing strategies are essential. One potential strategy is the integration of a clinical pharmacist into the inpatient team for daily interdisciplinary ward rounds. However, this approach remains under-researched in the context of inpatients with mental disorders.

**Objectives:**

This study aimed to evaluate the impact of a clinical pharmacist on medication-related problems (DRPs) focused on benzodiazepine and zolpidem use during daily ward rounds within an interdisciplinary team in a Slovenian psychiatric hospital.

**Methods:**

A retrospective observational pre-post study was conducted at Ormož Psychiatric Hospital in Slovenia, including patients treated between 2019 and 2020. During this study, clinical pharmacists provided recommendations focused on benzodiazepines and zolpidem. The primary outcomes assessed were the difference in the total number of DRPs observed at the time of hospital discharge compared to admission. The secondary outcomes evaluated adherence to existing treatment guidelines.

**Results:**

The study involved 20 participants with a mean age of 57.2 years (SD = 17.1). A total of 23 recommendations related to DRPs associated with benzodiazepine and zolpidem use were performed (1.15 per patient). Of these, 19 DRPs (82.6%) were identified as potential issues, while 4 DRPs (17.4%) were already expressed. Most DRPs concerning benzodiazepines and zolpidem were classified as unnecessary treatment, with 18 recommendations (78.3%). The remaining five recommendations (21.7%) addressed treatment effectiveness. The most common recommendation was the discontinuation of benzodiazepine and zolpidem therapy, suggested in 12 cases (56.5%), followed by adjustments to the treatment regimen, predominantly dose reductions, in 9 cases (39.1%). In only one case (4.3%) was the recommendation to initiate benzodiazepine treatment. Initially, the acceptance rate of recommendations was 100.0% (N = 23) at the time of discharge, but this decreased to 82.6% (N = 19) three months after discharge. Adherence to treatment guidelines improved (p < 0.05).

**Conclusions:**

The results indicate that this approach led to fewer DRPs, improved adherence to treatment guidelines, and a high acceptance rate. This is the first retrospective pre-post study in the European Union to include this collaboration in daily rounds at psychiatric hospitals, focusing on these medications. However, the study has notable limitations (non-randomized design and small sample size), which should be addressed in future research.

**Disclosure of Interest:**

M. Stuhec Grant / Research support from: Matej acknowledges the financial support from the Slovenian Research and Innovation Agency (research core funding No. J3-50127)., A. Gazdag: None Declared, Z. Cuk: None Declared, R. Oravecz: None Declared, B. Batinić: None Declared

